# Draft Whole-Genome Assemblies of Drug-Resistant Clinical Isolates of *Klebsiella pneumoniae* from the Philippines

**DOI:** 10.1128/genomeA.00475-17

**Published:** 2017-07-13

**Authors:** Marylette B. Roa, Veni R. Liles, Brian Carlmichael Torres, David Charles Klinzing, Evelina Lagamayo, Josephine Navoa-Ng, Maria Luisa G. Daroy

**Affiliations:** a St. Luke’s College of Medicine–William H. Quasha Memorial, Quezon City, Philippines; b Core Facility for Bioinformatics, Philippine Genome Center, University of the Philippines, Diliman, Quezon City, Philippines; c Research and Biotechnology, St. Luke’s Medical Center, Quezon City, Philippines; d Institute of Pathology, St. Luke's Medical Center, Quezon City, Philippines; e Infection Control Service, St. Luke’s Medical Center, Quezon City, Philippines

## Abstract

Here, we report the draft assemblies of 11 clinical isolates of *Klebsiella pneumoniae* that are resistant to cephalosporins, carbapenems, and/or colistin. The assemblies ranged from 5.37 Mbp to 5.70 Mbp in size. Several plasmid sequences were present, and resistance genes spanning multiple classes of antibiotics were predicted.

## GENOME ANNOUNCEMENT

Resistance against carbapenems, which are considered last-resort antibiotics, has been on the rise in the past decade (1), and resistance against colistin, an antibiotic used to treat carbapenem-resistant cases, has also been reported (2). While the mechanisms are diverse, the most common modes of resistance involve the production of antibiotic-degrading enzymes, modifications in the bacterial cell membrane, or through mutations (3).

Eleven clinical isolates of *Klebsiella pneumoniae* were collected during a passive surveillance of antimicrobial resistance in two branches of a teaching hospital in metropolitan Manila, Philippines (4). Nine of these isolates (ARPG-315, ARPG-340, ARPG-372, ARPG-379, ARPG-380, ARPG-381, ARPG-489, ARP-657, and ARP-664) were resistant to the carbapenems ertapenem, imipenem, and meropenem, of which two (ARP-657 and ARP-664) were also resistant to colistin. The two other isolates (ARPG-281 and ARPG-318) were susceptible to all three carbapenems, as well as colistin, but resistant to other antibiotics, including second-, third-, and fourth-generation cephalosporins. These two were isolated from the same patients from whom carbapenem-resistant *K. pneumoniae* strains were subsequently isolated. To further investigate the genetic basis of resistance in these isolates, whole-genome sequencing was performed.

Total DNA and plasmid DNA were extracted using the Wizard SV genomic DNA purification system (Promega Corporation) and PhasePrep BAC DNA kit (Sigma-Aldrich), respectively, according to the manufacturer’s protocol. Libraries were prepared separately for genomic and plasmid DNA using the Nextera library preparation kit (Illumina). Sequencing was carried out on an Illumina MiSeq platform and was performed twice for some samples to increase coverage. DNA and plasmid sequences were combined during analyses. FastQC version 0.11.2 (http://www.bioinformatics.babraham.ac.uk/projects/fastqc), and TrimGalore! version 0.3.8 (http://www.bioinformatics.babraham.ac.uk/projects/trim_galore) were used to preprocess the reads, while SPAdes version 3.5.0 (5, 6) was used for *de novo* assembly. CONTIGuator version 2.7 (7) was utilized to order the assemblies along the reference strain *K. pneumoniae* KPNIH31 sequence (accession no. NZ_CP009876) (8). Only contigs with ≥1 kbp length were used for subsequent analysis. For annotation, the NCBI Prokaryotic Genome Annotation Pipeline (PGAP) (http://www.ncbi.nlm.nih.gov/genomes/static/Pipeline.html) (9) was used.

Details on the assembly statistics are presented in Table 1. Sequencing coverage ranged from 47.78 to 131.35×, while assembly sizes were from 5.37 Mbp to 5.70 Mbp. The number of coding sequences (CDSs), rRNAs, and tRNAs annotated were, on average, 5,501, 15, and 77, respectively. *In silico* multilocus sequence typing (MLST) through the Pasteur MLST server (http://bigsdb.web.pasteur.fr) classified the isolates into five clonal groups (sequence type 1 [ST-1], ST-147, ST-273, ST-392, and ST-656), while plasmid typing using PlasmidFinder version 1.2 (https://cge.cbs.dtu.dk/services/PlasmidFinder) revealed multiple plasmid sequences. Furthermore, ResFinder version 2.1 (https://cge.cbs.dtu.dk/services/ResFinder) was able to predict resistance genes against different classes of antibiotics, including known carbapenemase genes *bla*_NDM-1_ in two carbapenem-resistant isolates (ARP-657 and ARP-664) and *bla*_NDM-7_ in five carbapenem-resistant isolates (ARPG-340, ARPG-372, ARPG-379, ARPG-380, and ARPG-381). No carbapenemase genes were annotated in the assemblies of the carbapenem-resistant isolates ARPG-315 and ARPG-489. Meanwhile, no colistin resistance genes were found in the colistin-resistant isolates ARP-657 and ARP-664.

**TABLE 1:** Assembly statistics and annotations of the 11 multiple-drug-resistant *Klebsiella pneumoniae* clinical isolates

Isolate	Genome coverage (×)	Assembly size (bp)	No. of contigs	No. of CDSs, no. of rRNAs, no. of tRNAs	No. of MLSTs	Plasmid types	Resistance genes	Accession no.
ARPG-281	52.19	5,399,126	88	5,363, 9, 77	1	IncFIB(K), IncR, IncFII(K)	*aac(3)-IId*, *aac(6ʹ)Ib-cr*, *aph(3ʹ)-Ia*, *ARR-3*, *bla*_DHA-1_, *bla*_OXA-1_, *bla*_SHV-1_, *catB3*, *fosA*, *oqxA*, *oqxB*, *qnrB4*, *sul1*	NBOH00000000
ARPG-315	107.55	5,372,623	43	5,282, 9, 77	1	IncFIB(K), IncR, IncFII(K)	*aac(3)-IId*, *aac(6ʹ)Ib-cr*, *ARR-3*, *bla*_DHA-1_, *bla*_OXA-1_, *bla*_SHV-1_, *catB3*, *dfrA1*, *fosA*, *oqxA*, *oqxB*, *qnrB4*, *sul1*, *tet*(A)	NBOI00000000
ARPG-318	67.8	5,395,829	53	5,286, 19, 77	147	IncR, IncFIA(HI1)	*aac(3)-IIa*, *aac(6ʹ)Ib-cr*, *bla*_CTX-M-15_, *bla*_OXA-1_, *bla*_SHV-11_, *bla*_TEM-1B_, *catB3*, *dfrA14*, *fosA*, *oqxA*, *oqxB*, *qnrB66*, *strA*, *strB*, *sul2*, *tet*(A)	NBOJ00000000
ARPG-340	131.35	5,662,273	89	5,685, 20, 76	273	IncHI1B, IncA/C2, IncFIB(Mar), IncX3	*aac(3)-IId*, *aac(6ʹ)Ib-cr*, *aadA5*, *aph(3′)-Ic*, *bla*_CTX-M-15_, *bla*_NDM-7_, *bla*_OXA-1_, *bla*_SHV-11_, *bla*_TEM-1B_, *catB3*, *dfrA17*, *erm*(42), *floR*, *fosA*, *mph*(A), *oqxA*, *oqxB*, *qnrS1*, *sul1*	NBOK00000000
ARPG-372	68.02	5,704,983	181	5,754, 16, 77	273	IncHI1B, IncA/C2, IncFIB(Mar), IncX3	*aac(3)-IId*, *aac(6ʹ)Ib-cr*, *aadA5*, *aph(3ʹ)-Ic*, *bla*_CTX-M-15_, *bla*_NDM-7_, *bla*_OXA-1_, *bla*_SHV-11_, *bla*_TEM-1B_, *catB3*, *dfrA17*, *erm*(42), *floR*, *fosA*, *mph*(A), *oqxA*, *oqxB*, *qnrS1*, *sul1*	NBOL00000000
ARPG-379	68.04	5,631,243	67	5,613, 19, 77	273	IncHI1B, IncA/C2, IncFIB(Mar), IncX3	*aac(3)-IId*, *aac(6ʹ)Ib-cr*, *aadA5*, *aph(3ʹ)-Ic*, *bla*_CTX-M-15_, *bla*_NDM-7_, *bla*_OXA-1_, *bla*_SHV-11_, *bla*_TEM-1B_, *catB3*, *dfrA17*, *erm*(42), *floR*, *fosA*, *mph*(A), *oqxA*, *oqxB*, *qnrS1*, *sul1*	NBOM00000000
ARPG-380	58.73	5,623,615	75	5,610, 19, 77	273	IncHI1B, IncA/C2, IncFIB(Mar), IncX3	*aac(3)-IId*, *aac(6ʹ)Ib-cr*, *aadA5*, *bla*_CTX-M-15_, *bla*_NDM-7_, *bla*_OXA-1_, *bla*_SHV-11_, *bla*_TEM-1B_, *catB3*, *dfrA17*, *erm*(42), *floR*, *fosA*, *mph*(A), *oqxA*, *oqxB*, *qnrS1*, *sul1*	NBON00000000
ARPG-381	96.98	5,664,647	73	5,668, 17, 76	273	IncHI1B, IncA/C2, IncFIB(Mar), IncX3	*aac(3)-IId*, *aac(6ʹ)Ib-cr*, *aadA5*, *aph(3ʹ)-Ic*, *bla*_CTX-M-15_, *bla*_NDM-7_, *bla*_OXA-1_, *bla*_SHV-11_, *bla*_TEM-1B_, *catB3*, *dfrA17*, *erm*(42), *floR*, *fosA*, *mph*(A), *oqxA*, *oqxB*, *qnrS1*, *sul1*	NBOO00000000
ARPG-489	59.31	5,521,319	122	5,515, 18, 76	392	IncFIB(K), IncN, IncFII(K)	*aac(6ʹ)Ib-cr*, *bla*_CTX-M-15_, *bla*_OXA-1_, *bla*_SHV-12_, *bla*_TEM-1B_, *catB3*, *fosA*, *oqxA*, *oqxB*, *qnrB66*, *strA*, *strB*, *sul2*, *tet*(A)	NBOP00000000
ARP-657	114.78	5,460,619	74	5,373, 12, 77	656	IncHI1B, IncFIB(Mar)	*aac(6ʹ)Ib-cr*, *aadA2*, *armA*, *bla*_CTX-M-15_, *bla*_NDM-1_, *bla*_OXA-1_, *bla*_SHV-1_, *catA1*, *catB3*, *dfrA12*, *fosA*, *mph*(E), *msr*(E), *oqxA*, *oqxB*, *qnrB66*, *sul1*	NBOQ00000000
ARP-664	47.78	5,454,384	79	5,364, 10, 78	656	IncHI1B, IncFIB(Mar)	*aac(6ʹ)Ib-cr*, *aadA2*, *armA*, *bla*_CTX-M-15_, *bla*_NDM-1_, *bla*_OXA-1_, *bla*_SHV-1_, *catA1*, *catB3*, *dfrA12*, *fosA*, *mph*(E), *msr*(E), *oqxA*, *oqxB*, *qnrB66*, *sul1*	NBOR00000000

### Accession number(s).

The whole-genome sequences have been deposited at DDBJ/ENA/GenBank under the accession numbers listed in Table 1. The versions described in this paper are the first versions.
